# Intravoxel incoherent motion diffusion MR and diffusion kurtosis imaging for discriminating atypical bone metastasis from benign bone lesion

**DOI:** 10.1259/bjr.20190119

**Published:** 2019-06-06

**Authors:** Gang Wu, Ruyi Xie, Xuanlin Liu, Bowen Hou, Yitong Li, Xiaoming Li

**Affiliations:** 1 Department of Radiology, Tongji Hospital, Tongji Medical College, Huazhong University of Science and Technology, Wuhan, Hubei Province, China

## Abstract

**Objectives:**

To investigate the feasibility of intravoxel incoherent motion (IVIM) diffusion MR and diffusion kurtosis imaging (DKI) in discriminating atypical bone metastasis from benign bone lesion in patients with tumors.

**Methods:**

Patients with bone lesions in lower extremity suspected of metastases were enrolled in this prospective study. IVIM diffusion MR and DKI were performed before biopsy. Apparent diffusion coefficient (ADC), true diffusion (D), perfusion fraction (f) and perfusion-related pseudodiffusion (D*) were generated with IVIM, while mean kurtosis (MK) and mean diffusion (MD) generated with DKI. Two radiologists blinded to pathology results separately measured these parameters for each lesion through drawing region of interest. Intraclass correlation coefficient was used to determine the inter-reader viability in measurement. The patients with pathology-confirmed metastasis or benign lesion were analyzed. The Mann–Whitney test was used to compare IVIM and DKI parameters between metastasis group and benign lesion group. Receiver operating characteristic curves were constructed to evaluate the ability of discrimination.

**Results:**

Bone lesions from 28 patients (metastasis, *n* = 15; benign lesion, n = 13; mean age = 55 years; age range, 34~77) were analyzed with IVIM and DKI. Intraclass correlation coefficient was greater than 0.8 for all parameters. ADC, D and MD were significantly lower in metastases versus benign lesions (*p﻿*<0.05). MK and f value were significantly higher in metastases versus benign lesions (*p*<0.05). D* was not significantly different between the two groups (*p*>0.05). Areas under curve for ADC, D, f, MK and MD were 0.935, 0.939, 0.891, 0.840 and 0.844 respectively.

**Conclusions:**

IVIM and DKI derived parameters distinguish between atypical bone metastasis and benign bone lesion in selected patients with tumors.

**Advances in knowledge:**

Bone metastasis and benign bone lesion differ in water molecular diffusion.

Intravoxel incoherent motion derived true diffusion distinguishes between atypical bone metastasis and benign lesion.

## Introduction

It is of great importance to discriminate bone metastasis from benign bone lesion in patients with primary tumors, as the treatment paths differ greatly.^1–3^ CT and MR can identify typical bone metastasis, such as multiple metastases. However, conventional CT and MR are poor in distinguishing between atypical bone metastasis and benign bone lesion, because they have similar image characteristics. Bone biopsy is the gold standard for identification of metastasis.^4^ However, bone biopsy was not widely used for this purpose in clinical practice due to invasiveness. Noninvasive diagnositic techniques that could discriminate atypical metastasis from benign lesion were urgently required.

Cell density differs between benign lesion and malignancy, so could be used for discrimination of bone metastasis and benign bone lesion. Diffusion-weighted imaging (DWI) derived apparent diffusion coefficient (ADC) can reflect cell density, and was reported to distinguish between malignancy and benign lesion.^5,6^ However, conventional DWI-derived ADC involves both water molecule diffusion and microcirculation perfusion. ADC should be modified so as to accurately reflect cell density. Intravoxel incoherent motion (IVIM) diffusion MR was developed to separate diffusion from perfusion.^7^ By using multiple *b* values of less than 200 s/mm^2^, true diffusion (D) and perfusion-related pseudodiffusion (D*) can be separately obtained. D was reported to perform better than ADC in distinguishing between benign lesion and malignancy.^2^

It is well established diffusion deviates from Gauss distribution in tissues of complex structure. Diffusion kurtosis imaging (DKI) was thus raised to deal with this non-Gauss diffusion.^8^ Mean kurtosis (MK) and mean diffusion (MD) can be generated with DKI. MK is correlated with tissue complexity, which differs between benign lesion and malignancy.

IVIM and DKI were used for evaluating tumor in plenty of studies.^2,9–11^ However, there are few publications using IVIM or DKI to discriminate atypical bone metastasis from benign bone lesion. The purpose of the study is therefore to investigate the feasibilty of IVIM and DKI in the discrimination of bone metastasis and benign bone lesion in selected patients with tumors.

## METHODS and Materials

### Patients

This is a prospective study approved by the university Institutional Review Broad. Informed consent was obtained from each patient before the study. Inclusion criteria: (1) patients had primary tumors; (2) CT or MR identified bone lesions in lower extremity. Exclusion criteria: (1) typical bone metastasis (such as multiple metastases); (2) standard contraindications to MR (such as claustrophobia); (3) biopsy pathology not available. From July 2015 to June 2018, lower extremity bone lesions were identified in 157 patients with tumors. Exclusion of patients was as follows: typical metastases (*n* = 114); contraindications to MR (*n* = 4); pathology result unavailable (*n* = 11). Thus, 28 out of 157 cases were finally analyzed.

All MR examinations were performed at a 3.0 T whole body scanner (Siemens, Skyra, Enlargen, Germany). An 18-element body coil was used to cover the region of interest. Fast spin echo (FSE) *T*_2_ was performed in saggital plane for localizing bone lesion with the following parameters: repetition time (TR), 2800 ms; echo time (TE), 92 ms; thickness, 4 mm; number of slice, 14 or more; matrix, 384 × 192; Grappa = 2. IVIM diffusion MR was performed in axial plane with the following parameters: TR, 3000 ms; TE, 61 ms; field of view (FOV), 20 × 20 cm or bigger to fit of leg size; thickness, 4 mm; number of slice, 14 or more; matrix, 140 × 140; Grappa = 2; directions, 3; *b* = 0, 10, 20, 30, 40, 50, 75, 100, 150, 200, 400, 700, 1000, 1500 s/mm^2^. DKI was performed in axial plane with the following parameters: TR, 3370 ms; TE, 68 ms; FOV, 20 × 20 cm or bigger to fit of leg size; thickness, 4 mm; number of slices, 14 or more; matrix, 140 × 140; Grappa = 2; directions, 3; *b* = 0, 100, 700, 1400, 2100 s/mm^2^.

### Data analysis

The source data were transferred to a dedicated workstation with a software (DKI_tool_3.4). This software generated MK map and MD map for DKI data, and ADC map, D map, D* map and perfusion fraction (f) map for IVIM data. Region of interest (ROI) of different areas were drawn on *b* = 0 map first and then copied to other parameter maps. ROI were drawn on three consecutive slices where the lesion size was the largest. Values from the three slices were averaged.

### Biopsy and pathology

Bone biopsy was performed for 28 patients who underwent IVIM and DKI. None of the 28 cases was primary malignancy. Metastasis was confirmed in 15 out of 28 cases. The other 13 cases were all benign: fibrous dysplasia, *n* = 3; endophytic chondroma, *n* = 1; simple bone cyst, *n* = 2; inflammatory granuloma, *n* = 3; nodular hyperplastic hematopoietic bone marrow, *n* = 4.

### Statistical analysis

SPSS 22.0 was used to perform all statistical analysis. The Mann–Whitney test was used to compare IVIM and DKI parameters between bone metastases and benign bone lesions. Receiver operating characteristic (ROC) curve was constructed to evaluate the ability of discrimination. The non-paired student’s *t* test was used to compare patient age between two groups. The χ^2^ test was used to identify gender difference. *p-*value less than 0.05 was considered statistically significant.

## Results

From July 2015 to June 2018, 28 patients (mean age = 55 years; age range, 34~77) with primary tumors (breast cancer, *n* = 4; prostatic cancer, *n* = 4; gastric cancer, *n* = 5; colon cancer, *n* = 3; lung cancer, *n* = 5; liver cancer, *n* = 4; renal carcinoma, *n* = 2; oophoroma, *n* = 1) and bone lesions suspected of metastases underwent IVIM diffusion MR, DKI and bone biopsy. The pathology result was metastasis in 15 cases, while benign bone lesion in the other 13 cases.

There is no significant difference in age or gender (*p* ＞ 0.05) between bone metastasis group (mean age = 55 years, male: female = 9: 6) and benign lesion group (mean age = 56 years, male: female = 8: 5). IVIM and DKI parameters of benign lesion and metastasis are shown in Table 1. ADC, D and MD were significantly lower in metastases versus benign lesions (*p* ＜ 0.05, see Table 1). MK and f were significantly higher in metastases *vs* benign lesions (*p* ＜ 0.05, see Table 1). There is no significant difference in D* between the two groups (*p* ＞ 0.05, see Table 1).

**Table 1.** IVIM and DKI parameters were compared between benign bone lesion and bone metastasis using a Mann–Whitney test

	Benign lesion (*n* = 13)	Metastasis (*n* = 15)
ADC (×10^-3 ^mm^2^/s)	1.95 ± 0.39	1.23 ± 0.27	＜0.001
D (×10^-3 ^mm^2^/s)	1.78 ± 0.42	1.12 ± 0.22	0.005
D^* ^(×10^-3 ^mm^2^/s)	6.86 ± 3.53	9.72 ± 4.89	0.168
f (%)	3.43 ± 2.99	10.0 ± 3.98	＜0.001
MK	0.36 ± 0.22	0.76 ± 0.45	0.03
MD (×10^-3 ^mm^2^/s)	1.91 ± 0.53	1.26 ± 0.46	0.004

ADC, apparent diffusion coefficient; D*, perfusion-related pseudo diffusion; D, true diffusion; DKI, diffusion kurtosisimaging; IVIM, intravoxel incoherentmotion; MD, mean diffusion; MK, mean kurtosis; f, perfusion fraction.

AUC and 95% confidence interval for ADC, D, f, D^*^, MK and MD in discrimination of metastasis and benign lesion are shown in Table 2. D had the highest AUC among all parameters, followed by ADC. AUC was higher in IVIM parameters except D^*^ *vs* DKI parameters.

**Table 2.** AUC and 95% confidence interval for IVIM and DKI parameters in discriminating bone metastasis from benign bone lesion

	AUC	95% CI
ADC	0.935	0.774–0.993
D	0.939	0.779–0.994
f	0.891	0.716–0.977
D^*^	0.701	0.499–0.858
MK	0.84	0.653–0.950
MD	0.844	0.657–0.952

ADC, apparent diffusion coefficient; AUC, area under curve; CI, confidence interval; D*, perfusion-related pseudo-diffusion; D, true diffusion; DKI, diffusion kurtosis imaging; IVIM, intravoxel incoherentmotion; MD, mean diffusion; MK, mean kurtosis; f, perfusion fraction.

**Figure 1.**
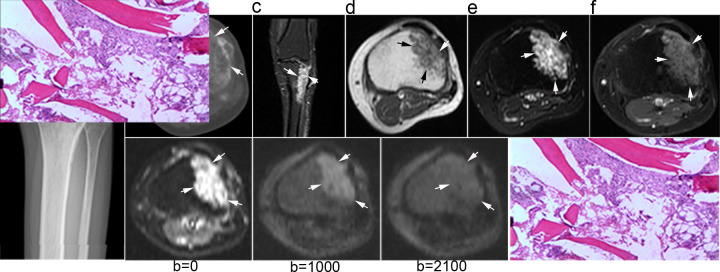
Female, 45 years, breast cancer. The X-ray showed abnormal structure of bone trabecula at lateral tibial plateau (a, arrows). Osteoporosis and periosteal hyperplasia could be seen on transverse CT image (b, arrows). Coronal fat-suppressed *T*_2_ image displayed a lesion of hyperintensity at proximal tibia (c, arrows). Fast spin echo *T*_1_ (d), *T*_2_ (e), and contrast-enhanced *T*_1_ (f) could display the border of the lesion (d, e, f, arrows) better than X-ray or CT (a, b, arrows). It was difficult to determine whether this lesion was metastasis with conventional images only. The lesion was very high signal on *b* = 0 image from diffusion kurtosis imaging, and brighter than background on *b* = 1000 from intravoxel incoherent motion diffusion MR. It was isointensity on *b* = 2100 image where the border of lesion could not be identified. Thus diffusion kurtosis imaging did not support the diagnosis of metastasis. This lesion was confirmed not a metastasis from breast cancer by pathology.

**Figure 2.**
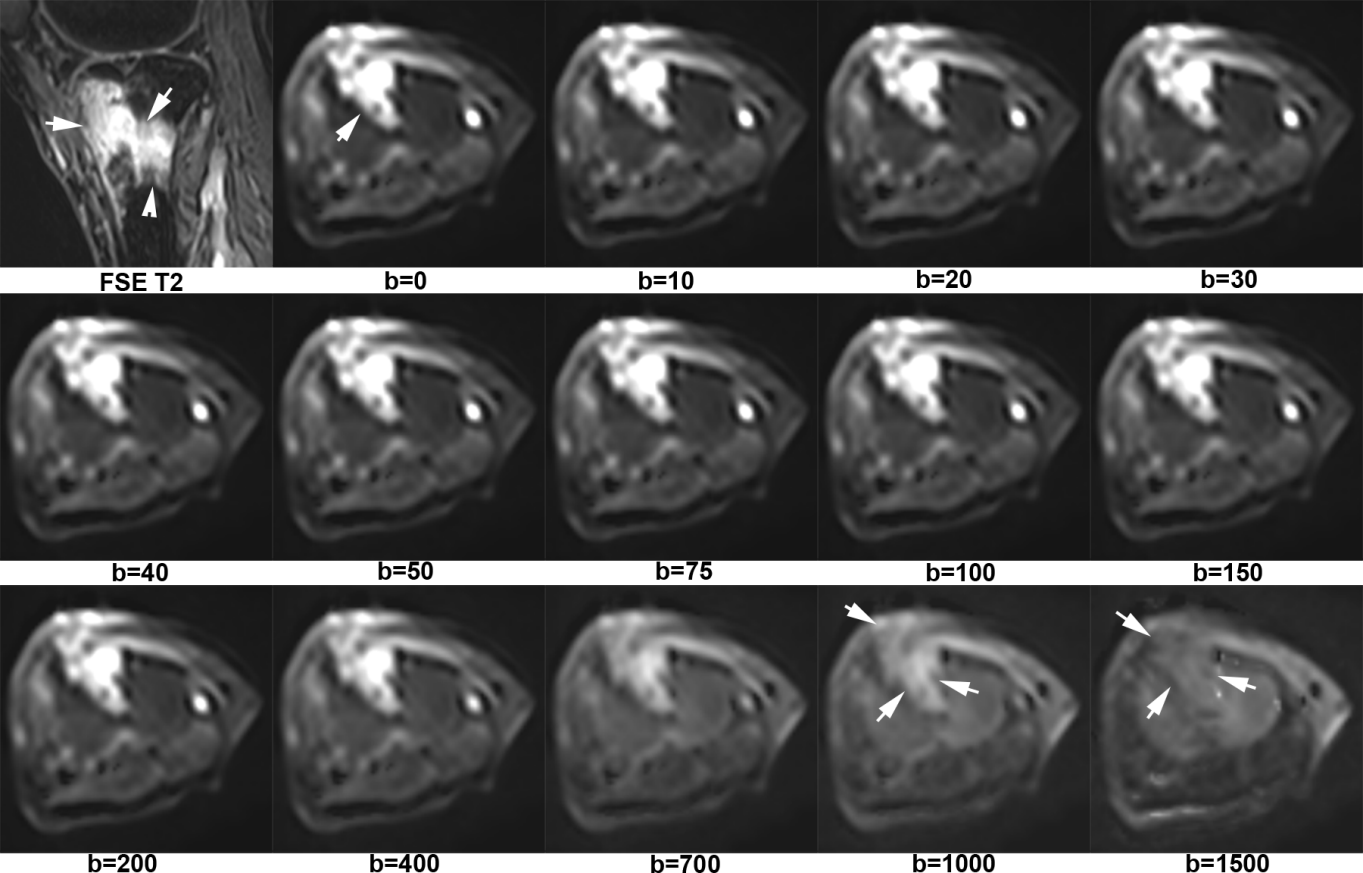
A bone lesion of hyperintensity was identified at tibia of a patient with gastric cancer. It was very high signal when *b*-value was less than 200. The intensity of the lesion decreased with the increase of *b*-value. On b=1500 map, this lesion was isointensity. It was an inflammatory granuloma confirmed by pathology.

**Figure 3.**
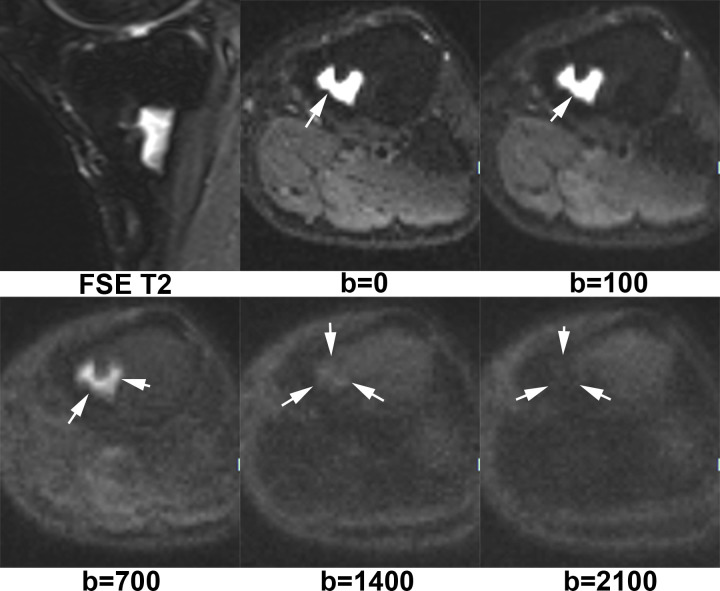
A lesion of hypertensity was displayed with FSE *T*_2_ image at tibia of a patient with lung cancer. On *b* = 0 and *b* = 100 maps, this lesion was very high intensity. The signal intensity of the lesion decreased with the increase of *b*-value. This lesion was isointensity on *b* = 1400 map, while hypointensity on *b* = 2100 map. It was a nodular hyperplastic hematopoietic bone marrow confirmed with pathology. FSE, fast spin echo.

**Figure 4.**
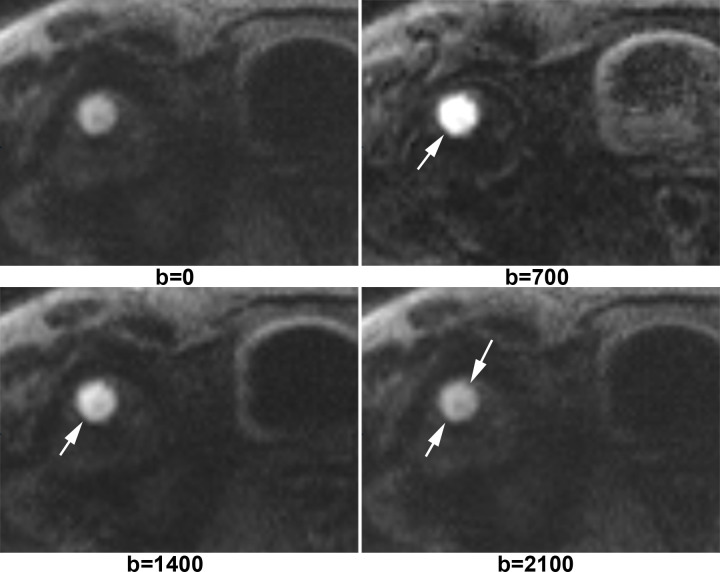
A circular lesion was identified at right femoral head of a patient with breast cancer. This lesion was very high intensity on *b* = 0, *b* = 700 and *b* = 1400 maps. It was still hyperintensity on *b* = 2100 map. Biopsy pathology confirmed it was a metastasis from breast cancer.

**Figure 5.**
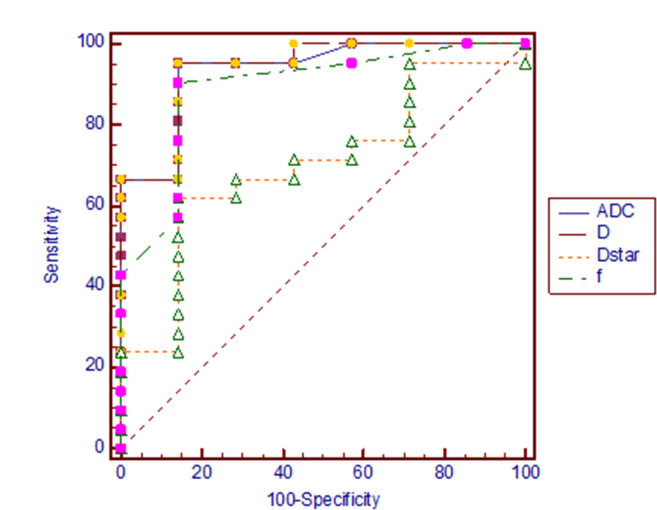
ROC curves for IVIM parameters in distinguishing between bone metastasis and benign bone lesion. AUC of ADC, D, f and D^*^ were respectively 0.935, 0.939, 0.891 and 0.701. ADC, apparent diffusion coefficient; AUC, areas under curve; IVIM, intravoxel incoherent motion; ROC, receiver operating characteristic.

**Figure 6.**
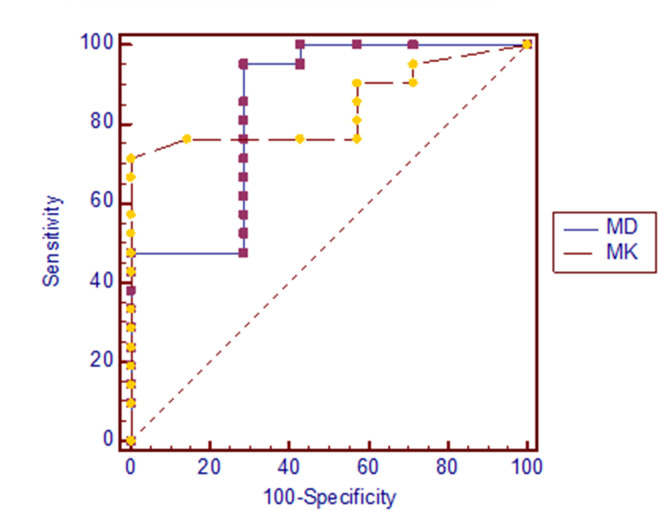
ROC curves for DKI parameters in distinguishing between bone metastasis and benign bone lesion. AUC of MK and MD were respectively 0.840 and 0.844. AUC, areas under curve; DKI, diffusion kurtosis imaging; MD, mean diffusion; MK, meankurtosis; ROC, receiver operating characteristic.

Figure 1 shows X-ray, CT, conventional MR and diffusion weighted MR images for a case suspected of metastasis. IVIM source images are shown in Figure 2. DKI source images are shown in Figures 3 and 4. Figures 5 and 6 show ROC curves for IVIM and DKI parameters respectively.

## Discussion

The feasibility of IVIM diffusion MR and DKI in discriminating atypical bone metastasis from benign bone lesion was investigated in the study. The most important findings were: (1) atypical metastasis and benign lesion differed in ADC, D, f, MD and MK; (2) IVIM derived D value was the best parameter for identification of metastasis.

By using multiple *b﻿*-values of less than 200 s/mm^2^, IVIM can separate true diffusion from perfusion-related pseudodiffusion, which is blood movement in capillaries.^12^ True diffusion is described with D, while pseudodiffusion is described with D*. Perfusion fraction generated with IVIM is discribed with f. The f correlates with the abundance of capillaries in tissue. Thus, both D* and f reflect tissue vascularization. Traditional DWI is based on Gauss distribution, while DKI based on non-Gauss distribution. DKI can reflect tissue microstructure more accurately.^13^ DKI derived MK reflects diffusion heterogeneity that correlates with tissue complexity.

We found ADC, D and MD were lower in metastases *vs* benign lesions. The most possible explanation is metastasis has greater cell density than benign lesion. The extracellular space was samller in metastasis *vs* benign lesion, so water molecular diffusion was more restricted in metastasis. AUC for ADC, D and MD were all above 0.8, so these parameters seemed suitable for discrimination of atypical metastasis and benign lesion. We found AUC was higher in D *vs* ADC. The most possible explanation is D has eliminated the influence of perfusion, while ADC not. D is more suitable than ADC to reflect cell density, especially for tussues of high vascularization. D seemed better than ADC in discrminating metastasis from benign lesion.

Most authors considered it necessary to use IVIM diffusion MR instead of conventional DWI for well-vascularized tissues.^14,15^ Metastases are highly vascularized in most cases, so IVIM is required to separate true diffusion from perfusion. In fact, we found D* was higher than D by an order of magnitude in most of metastasis cases. In contrast, benign bone lesison seemed not well-vascularized, as perfusion fraction was small in most of benign cases. We found f value was higher in metastasis *vs* benign lesion. AUC of f was above 0.8, so perfusion fraction seemed also suitable for the discrimination. We found D* was lower in benign lesion *vs* metastases, but the difference was not statistically significant. AUC of D* was poor, so pseudo diffusion seemed not suitable for the discrimination.

The tissue structure of metastasis is generally complex due to high cell density, necrosis and hemorrhage, resulting in non-Gauss diffusion at high *b* values. In contrast, benign lesion has relatively simple tissue structure. Atypical metastasis and benign lesion also differ in tissue complexity. In fact, we found MK value of metastasis was significantly higher than that of benign lesion. AUC of MK was above 0.8, so mean kurtosis seemed suitable for the discrimination.

It is well established that the accuracy of ADC value could be improved if more *b*-values are used.^16^ IVIM diffusion MR used more *b-*values than DKI. That is why AUC is higher in IVIM-derived ADC *vs* DKI derived MD. D and f had higher AUC than MK, so seemed more suitable in distinguishing between atypical metastasis and benign lesion. Ture diffusion with the highest AUC seemed the best parameter for the discrimination.

Our study has several limitations. First, the sample size is small. Invasive bone biopsy was not widely used for atypical metastasis. We only collected 28 cases during nearly 3 years. Multicenter large-size studies are needed to validate our results. Second, benign lesion included multiple categories in this study, so is not homogeneous. Simple and direct comparison is required to be done in future, such as metastasis *vs* fibrous dysplasia. Third, we did not perform a comparison between typical metastasis and atypical metastasis. We focused on the comparison of atypical metastasis *vs* benign lesion, as they really have similar image characteristics in conventional CT and MR.

## Conclusion

In conclusion, IVIM diffusion MR and DKI distinguish between atypical bone metastasis and benign bone lesion. IVIM derived true diffusion is the best parameter for the discrimination.
